# SG-APSIC1073: Incidence and predictors of *Escherichia coli*–producing extended-spectrum beta-lactamase (ESBL-Ec) in Queensland, Australia, from 2010 to 2019: A population-based spatial analysis

**DOI:** 10.1017/ash.2023.71

**Published:** 2023-03-16

**Authors:** Weiping Ling, Angela Cadavid-Restrepo, Luis Furuya-Kanamori, Patrick N.A. Harris, David L. Paterson

**Affiliations:** University of Queensland, Brisbane, Australia; University of Queensland, Brisbane, Australia; University of Queensland, Brisbane, Australia; University of Queensland, Brisbane, Australia; University of Queensland, Brisbane, Australia

## Abstract

**Objectives:** The dissemination of *Escherichia coli*–producing extended-spectrum β-lactamase (ESBL-Ec) is evident in the community. In this population-based spatial analysis, we sought to describe the distribution of ESBL-Ec and to identify predictors of incidence in the community. **Methods:** The study population was defined as individuals with the ESBL-Ec isolate in Queensland, Australia, from 2010 to 2019. Annual choropleth maps and a global Moran index were constructed to describe ESBL-Ec distribution. Getis-Ord Gi* was performed to identify “hot spots” of statistical significance. Using demographic factors and incidence per postal area from 2016, multivariable analyses with or without spatially structured random effects were performed. **Results:** In total, 12,786 individuals with ESBL-Ec isolate were identified. The incidence rate increased annually from 9.1 per 100,000 residents in 2010 to 49.8 per 100,000 residents in 2019. The geographical distribution changed from random to clustered in 2014. Hot spots were more frequently identified in the Outback and Far North Queensland, where remote communities and hotter weather are prevalent. Multivariable spatial analysis suggests that communities with higher socioeconomic status (RR, 0.66; 95% CI, 0.55–0.79 per 100 units) and employment in the agricultural industry (RR, 0.79; 95% CI, 0.67–0.95 per 10%) were protective of lower ESBL-Ec incidence. After accounting for multiple demographic factors, the residual, structured, random-effects model indicated that hot spots were still detected in more remote communities but also in several city regions. **Conclusions:** The change in distribution of ESBL-Ec across Queensland suggests the presence of area-level specific risk factors that enhance spread in the community. Risk factors for spread appear different between remote and city settings, and future research should be tailored to understand the respective area-level risk factors. Factors such as local temperature, antibiotic consumption, and access to services should be validated. Future public health measures to reduce transmission should be focused on the identified hot spots.

